# Change in *Emiliania huxleyi* Virus Assemblage Diversity but Not in Host Genetic Composition during an Ocean Acidification Mesocosm Experiment

**DOI:** 10.3390/v9030041

**Published:** 2017-03-08

**Authors:** Andrea Highfield, Ian Joint, Jack A. Gilbert, Katharine J. Crawfurd, Declan C. Schroeder

**Affiliations:** 1 The Marine Biological Association, The Laboratory, Citadel Hill, Plymouth PL1 2PB, UK; ancba@mba.ac.uk (A.H.); ianjoi@mba.ac.uk (I.J.); 2 The Microbiome Centre, Department of Surgery, University of Chicago, Chicago, IL 60637, USA; gilbertjack@uchicago.edu; 3 Division of Bioscience, Argonne National Laboratory, 9700 South Cass Avenue, Argonne, IL 60439, USA; 4 Department of Biological Oceanography, NIOZ–Royal Netherlands Institute for Sea Research, P.O. Box 59, 1790 AB Den Burg, Texel, The Netherlands; kate.crawfurd@gmail.com

**Keywords:** *Emiliania huxleyi*, CO_2_, ocean acidification, climate change, *Coccolithovirus*, EhV

## Abstract

Effects of elevated *p*CO_2_ on *Emiliania huxleyi* genetic diversity and the viruses that infect *E. huxleyi* (EhVs) have been investigated in large volume enclosures in a Norwegian fjord. Triplicate enclosures were bubbled with air enriched with CO_2_ to 760 ppmv whilst the other three enclosures were bubbled with air at ambient *p*CO_2_; phytoplankton growth was initiated by the addition of nitrate and phosphate. *E. huxleyi* was the dominant coccolithophore in all enclosures, but no difference in genetic diversity, based on DGGE analysis using primers specific to the calcium binding protein gene (*gpa*) were detected in any of the treatments. Chlorophyll concentrations and primary production were lower in the three elevated *p*CO_2_ treatments than in the ambient treatments. However, although coccolithophores numbers were reduced in two of the high-*p*CO_2_ treatments; in the third, there was no suppression of coccolithophores numbers, which were very similar to the three ambient treatments. In contrast, there was considerable variation in genetic diversity in the EhVs, as determined by analysis of the major capsid protein (*mcp*) gene. EhV diversity was much lower in the high-*p*CO_2_ treatment enclosure that did not show inhibition of *E. huxleyi* growth. Since virus infection is generally implicated as a major factor in terminating phytoplankton blooms, it is suggested that no study of the effect of ocean acidification in phytoplankton can be complete if it does not include an assessment of viruses.

## 1. Introduction

The rise in anthropogenic CO_2_ in the atmosphere and subsequent dissolution in the oceans has changed the carbonate: bicarbonate: dissolved CO_2_ equilibrium, lowering seawater pH—a trend that is predicted to continue [1]. Change of pH is of particular significance for marine organisms that have calcium carbonate structures, such as corals and coccolithophores, because less alkaline conditions and pH-dependent shifts in equilibrium of the carbonate system will lead to higher dissolution rates of carbonate. Coccolithophores are ubiquitous and have global significance in regulating the carbon cycle in the oceans [2]. They form massive blooms, whose wide distribution and abundance is readily detected in satellite imagery. Given this wide distribution, it is important to determine if the lower pH of a future ocean will affect the success of coccolithophores and if there will be an impact on marine food webs and biogeochemical cycles.

The effect of changing pH on the important coccolithophore, *Emiliania huxleyi*, has been the focus of much research in recent years. However, results have been variable and consensus has been difficult to reach. In laboratory experiments, both negative and positive effects of increasing *p*CO_2_ have been described (see, for example, [3,4,5]). Another important approach has been to use large volume enclosures—mesocosms—to investigate a range of conditions that might apply to the future ocean. Unlike laboratory-based experiments, which usually focus on a single organism in the experimental design, mesocosms include all components of the pelagic system from viruses to zooplankton. By maintaining the possibility of complex interactions between different components of the food web, it has been assumed that mesocosms should offer advantages over single-organism culture experiments. However, results have also been rather variable. Early experiments suggested negative effects of higher *p*CO_2_ on production and calcification in *E. huxleyi* [6], but other studies have indicated that the effect of increased *p*CO_2_ is minimal for other coccolithophore species [7]. Time series analysis of natural populations has been another approach and a recent analysis of coccolithophore abundance in the North Sea concluded that increasing pCO_2_ on decadal scales has resulted in larger coccolithophore populations [8]. The contradictory results make it difficult to robustly predict how natural populations will respond to pH change in a future ocean.

We suggest that real understanding of the effect of pH change/higher *p*CO_2_ requires more detailed information than has been obtained to date, particularly in relation to phytoplankton genetic variability and virus infection. In this study, the response of a population of *E. huxleyi* to increased *p*CO_2_ at the early stages of a phytoplankton bloom in a mesocosm experiment has been investigated. In addition, changes in diversity of the viruses that infect *E. huxleyi* (EhVs) were followed during the experiment with diversity distinguished on the basis of a major capsid protein (*mcp*) gene as a molecular marker. Virus diversity is known to be high [9,10], and viruses are important components of the pelagic system that require attention in both laboratory and mesocosm experiments. All *E. huxleyi*-infecting viruses that have been characterised to date are dsDNA viruses, classified in the family *Phycodnaviridae*. They are significant mortality agents of *E. huxleyi*, implicated in the termination of large-scale blooms. Viruses have a proven role in structuring and maintaining host population diversity [11,12,13,14] and virus infection can have significance for the cycling of carbon and trace elements. The ‘viral shunt’ releases nutrients as well as dissolved and particulate organic matter from lysed organisms into the organic carbon pool [15,16]. This material, and the rate of supply by viral lysis, of substrates for heterotrophic microbial communities, has implications for species succession, biogeochemical cycles and feedback mechanisms. 

Given that diversity of both *E. huxleyi* and EhV assemblages can be variable, and that different *E. huxleyi* and EhV assemblages may come to eventually dominate natural communities, it is important to know the impact of elevated *p*CO_2_. *E. huxleyi* blooms are typically dominated by certain alleles/genotypes, and by asexual reproduction, with rarer alleles/genotypes tending to fluctuate [17]. As such, the impact of elevated CO_2_ on the composition of *E. huxleyi* populations can easily be monitored by studying these entities. Virus infection may be an explanation for some of the variability reported from different mesocosm experiments that were designed to investigate potential effects of higher *p*CO_2._ In this study, the aim was to understand how *p*CO_2_ change might influence *E. huxleyi* and EhV population structure and the diversity of host and virus. We suggest that viral infection can result in variability between replicate enclosures.

## 2. Materials and Methods

### 2.1. Experimental Set-Up and Sampling 

The mesocosm experiment was done in the Raunefjorden at the University of Bergen Espegrand field station, Norway (latitude: 60°16′ N; longitude: 5°13′ E) during May 2006. The experiment had two phases. The first phase, until 15 May, followed the development of a phytoplankton bloom and the second phase studied the decline of the bloom; only the first phase of the experiment is considered here. Six polyethylene enclosures of 2 m diameter and 3.5 m depth containing 11 m^3^ water were moored ca. 200 m from the shore and filled simultaneously with fjord water, salinity 31.4, and temperature 10.4 °C. Over a 40 h period from 4–6 May, 3 enclosures were bubbled with air enriched with CO_2_ to 760 ppmv whilst the other 3 enclosures were bubbled with air at ambient *p*CO_2_. The *p*CO_2_ in the air mixture was measured inline with a LI-COR 6262 CO_2_/H_2_O analyser (LI-COR, Inc., Lincoln, NE, USA). After equilibration, the pH of each of the high *p*CO_2_ treatments was 7.8 and the ambient treatment mesocosms were all pH 8.15. High precision alkalinity and *p*CO_2_ measurements were made throughout the experiment and pH was calculated [18]. All mesocosms were covered with UV-transparent polyethylene to maintain the appropriate CO_2_ concentration in the headspace above the enclosures, whilst allowing transmission of the complete spectrum of light and the exclusion of rainwater. Phytoplankton blooms were initiated on 6 May by the addition of 15 µmol·L^−1^ NaNO_3_ bringing the initial nitrate concentration to 16.1 µmol·N·L^−1^ and 1 µmol·L^−1^ NaH_2_PO_4_ to give an initial phosphate concentration 1.19 µmol·P·L^−1^. Silicate was not added because the aim was to test the effects of pH change on coccolithophores, but rather to stimulate diatom growth; the initial silicate concentration was 0.25 µmol·Si·L^−1^. 

### 2.2. Water Sampling

The majority of measurements were made on water samples taken at the same time each day, between 10 a.m. and 11 a.m. Water samples were collected in 5 L carboys and transported to the shore laboratory where they were processed in a temperature controlled room at ambient seawater temperature. 

### 2.3. Nutrient and Phytoplankton Analysis

Nutrient concentrations were determined on duplicate water samples by colorimetric analysis using the methods of Brewer and Riley [19] for nitrate, Grasshoff [20] for nitrite, and Kirkwood [21] for phosphate. Chlorophyll concentration was determined fluorometrically each day during the experiment, using the method of Holm-Hansen et al. [22] on water samples filtered through GFF glass fibre filters to monitor phytoplankton development. Samples were also taken for HPLC analysis of phytoplankton pigments, with GFF filters being stored at −80 °C between the period of sampling and laboratory analysis. Coccolithophore numbers were enumerated by analytical flow cytometry.

The rate of carbon fixation was estimated from the incorporation of ^14^C-bicarbonate following the method of Joint and Pomroy [23]. Surface water samples were collected from each mesocosm at dawn and transferred into five 60 mL clear polycarbonate bottles and a single black polycarbonate bottle; all bottles were cleaned following JGOFS protocols [24] to reduce trace metal contamination. Each bottle was inoculated with 37 kBq (1 µCi) NaH^14^CO_3_; bottles were incubated at the surface and depths of 0.5, 1, 2 and 3 m in the fjord adjacent to the mesocosm facility for 24 h. Samples were filtered through 0.2 µm pore-size polycarbonate filters, dried, and treated with fuming HCl to remove unfixed ^14^C and the assimilated ^14^C fraction was measured in a liquid scintillation counter. The efficiency of the LSC was determined with an external standard, channels ratio method. The quantity of ^14^C added to the experimental bottles was determined by adding aliquots of the stock ^14^C solution to a CO_2_-absorbing scintillation cocktail, which was counted immediately in the LSC.

### 2.4. Extraction of DNA

Collected water was stored at 4 °C until it was filtered, which occurred within several hours. Five litres of water from each mesocosm were filtered through a Sterivex-GP Sterile Vented Filter Unit, 0.22 µm (Millipore, Merck KGaA, Darmstadt, Germany). Filters were snap frozen in liquid nitrogen and maintained at −80 °C until they were processed. In addition, 2 mL 1× PBS was applied to the filters to wash off biomass and this was pelleted by centrifugation. DNA was extracted using the Qiagen DNeasy blood and tissue kit (Qiagen, Valencia, CA, USA) according to the manufacturer’s instructions.

### 2.5. Polymerase Chain Reaction (PCR) and Denaturing Gradient Gel Electrophoresis (DGGE) of E. huxleyi and EhV Populations

PCR/DGGE analyses of extracted DNA from the 6 mesocosms were carried out according to the protocol for *E. huxleyi* and *E. huxleyi* viruses (EhV), as detailed in Schroeder et al. [25] and Schroeder et al. [13], respectively, using primers specific to the calcium binding protein gene (*gpa*) for *E. huxleyi* and the major capsid protein (*mcp*) gene for EhV. PCR products for *gpa* and *mcp* were run on a 30%–50% denaturing gel according to Schroeder et al. in order to visualise the respective community structures [13]. DGGE profiles for EhV were analysed using Genetools (Syngene, Cambridge, UK) using rolling disk baseline correction and minimum peak detection; width 7, height 3, volume 2% and Savitsky–Golay filter 3 to discriminate and quantify different bands/peaks.

### 2.6. Statistical Analysis

Ambient and high CO_2_ multi-dimensional analysis (MDA) ordinations were calculated using Primer (v6) [26] using Bray–Curtis resemblance matrices produced from the DGGE profiles where bands were detected according to their migration distance down the tracks using Genetools (Syngene, Cambridge, UK). Principal component analysis (PCA) were calculated in Primer using all data obtained in the experiment to investigate which components might define differences/similarities between samples.

## 3. Results

### 3.1. Bloom Evolution—pH, Nutrients and Primary Production

**Figure 1:**
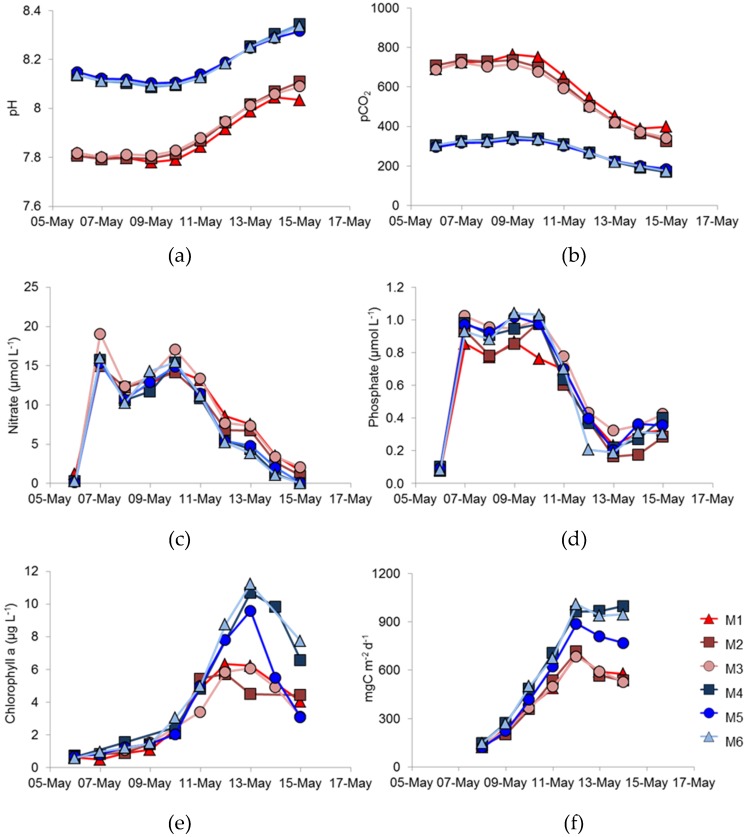
Temporal changes over the period of the experiment in (**a**) pH, which was calculated from measurements of (**b**) *p*CO_2_ in µatmospheres; (**c**) nitrate concentration, µmol·N·L^−1^; (**d**) phosphate concentration µmol·P·L^−1^; (**e**) chlorophyll concentration µg·L^−1^; and (**f**) depth-integrated primary production as mg·C·m^−2^·d^−1^. Enclosures M1 (▲), M2 (■), M3 (●), M4 (■), M5 (●), M6 (▲).

Following bubbling to achieve the target pHs in all mesocosms, the experimental phase was initiated on 6 May, by the addition of nitrate and phosphate. Initial pH of the non-modified treatment mesocosms was 8.14. Figure 1a shows the values of pH during the first nine days of the experiment that were calculated from high precision *p*CO_2_ data (Figure 1b) [18]. For four days, pH and *p*CO_2_ remained constant with little variation between replicate treatments. After 10 May, pH began to increase in all mesocosms, with declining *p*CO_2_ values as the phytoplankton bloom developed. Figure 1c,d record the changes in nitrate and phosphate concentration, including the initial nutrient addition. Both nutrients declined in concentration after 10 May as phytoplankton biomass increased (Figure 1e). Chlorophyll a concentration increased rapidly in all enclosures (Figure 1f), reaching a maximum on 13 May. However, there were differences in the maximum concentrations attained; the three high *p*CO_2_-treatment mesocosms had maximum chlorophyll concentrations of 6.23, 4.51 and 6.08 µg·L^−1^, but chlorophyll concentrations were higher (10.71 and 11.22 µg·L^−1^) in two of the ambient high *p*CO_2_-treatment mesocosms (4 and 6). A slightly lower phytoplankton biomass developed in enclosure M5—one of the ambient *p*CO_2_-treatment mesocosm—with a chlorophyll a concentration of 9.60 µg·L^−1^. The chlorophyll concentration in this mesocosm also declined more rapidly after 13 May than in the other treatments.

Primary production rates were very consistent in the three high *p*CO_2_-treatment mesocosms (Figure 1f), reaching maximum values on 12 May, with little variation between enclosures. In all the ambient *p*CO_2_ mesocosms, primary production was >900 mg C m^−2^·d^−1^ on 12 May and remained at this value for two days in M4 and M6. However, production in M5 was less than in the other two ambient *p*CO_2_-treatments, which is consistent with the lower chlorophyll concentration in this enclosure. 

### 3.2. E. huxleyi Genetic Composition during the Mesocosm Experiment 

*E. huxleyi* was a significant component of the phytoplankton assemblage that developed in each enclosure. Diatom numbers were insignificant because silicate was not added to the initial nutrient addition, being three orders of magnitude less abundant in light microscope analysis than the total flagellate fraction, which includes coccolithophores. Hopkins et al. [18] reported the dominance of large picoeukaryotes in each mesocosm assemblage but with the flagellates contributing greatest to phytoplankton biomass.

**Figure 2:**
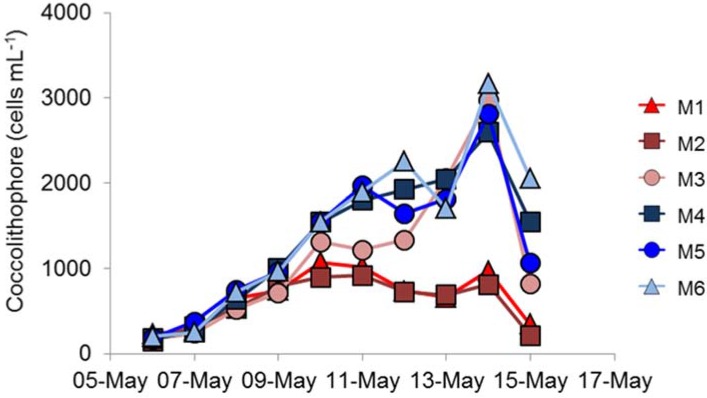
Total coccolithophore numbers assessed by flow cytometry. Enclosures M1 (▲), M2 (■), M3 (●), M4 (■), M5 (●), M6 (▲).

All enclosures showed steady increases in coccolithophore numbers (as assessed by flow cytometry) immediately after nutrient addition. Numbers reached 600–1000 cells mL^−1^ on 9 May, which is typical of numbers seen during the early-stages of *E. huxleyi* blooms (Figure 2) [12]. In the ambient-*p*CO_2_ treatments (M4, M5, M6) coccolithophore numbers increased until 12 May, but with a slight pause in growth, numbers increased further to a maximum of 2500–3000 cells mL^−1^ on 14 May. Cell numbers then declined to 1000–2000 cells mL^−1^ on 15 May. Coccolithophore biomass was different in the three replicate high *p*CO_2_-treatment mesocosms. In two enclosures, numbers plateaued at about 1000 cells mL^−1^, but, in the third mesocosm, numbers were higher and, indeed, very similar (3100 cells mL^−1^) to the peak biomass in the ambient *p*CO_2_-treatment mesocosms (Figure 2). Cell numbers declined in all six mesocosms after 14 May, even in those enclosures with lower cell numbers.

**Figure 3:**
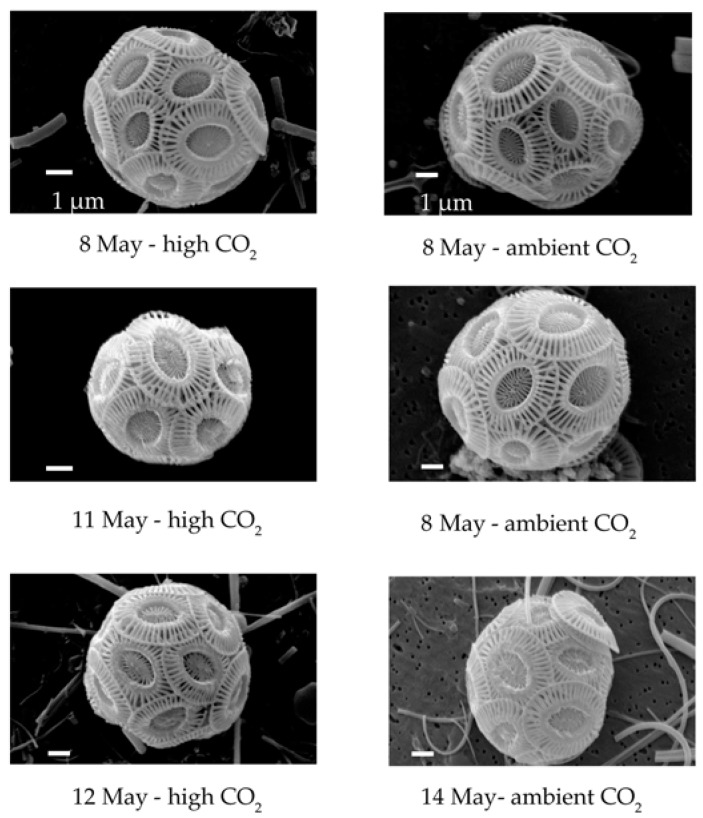
TEM images of identical *Emiliania huxleyi* morphologies (typical type A) present in both pCO_2_ treatments throughout the experiment.

Traditional microscopy, neither light nor electron, is capable of distinguishing between *E. huxleyi* genotypes with Figure 3 showing that identical morphology (typical type A) was present in both *p*CO_2_ treatments throughout the experiment. 

However, molecular analysis showed a large genetic diversity that was not revealed by microscopy. DGGE analysis of the *E. huxleyi* population using the *gpa* marker detected two to three dominant bands throughout the experiment as indicated by the arrows in Figure S1. This gene has been verified for *E. huxleyi* diversity analysis, with a limited number of genotypes known to exist [17] that can largely be separated by DGGE [25]. There was some small-scale variability in the *E. huxleyi* population between samples, as indicated by migration profiles. Overall, the *E. huxleyi* populations had similar genetic composition in all six mesocosms and no major differences were identified between treatments or replicates.

### 3.3. EhV Population Analysis 

**Figure 4:**
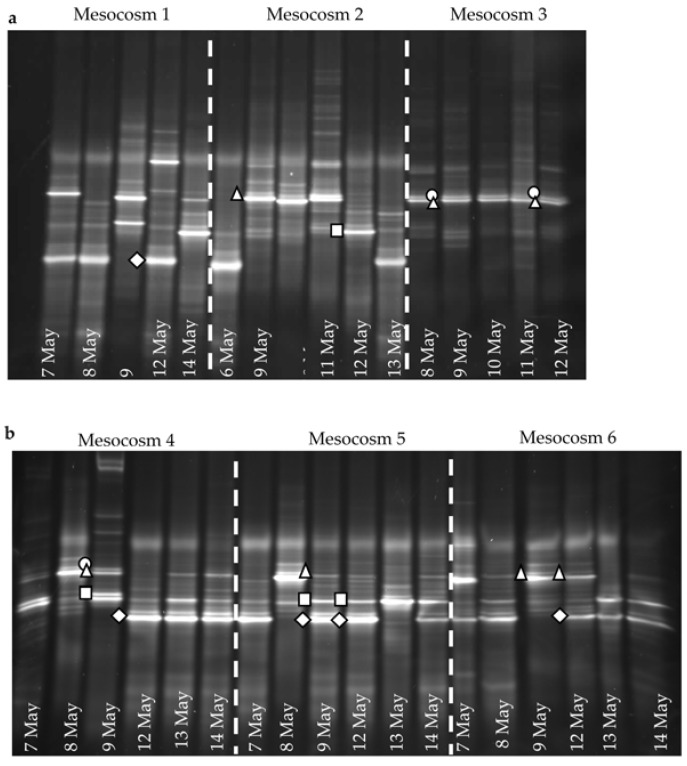
DGGE gels of EhV *mcp*-PCR products during the experiment from (**a**) high *p*CO_2_-treatment mesocosms, 1, 2, and 3 and (**b**) ambient *p*CO_2_-treatment mesocosms 4, 5, and 6. Bands that migrated at the same position when run on the same gel are indicated with the same symbol.

Flow cytometry revealed the presence of large DNA viruses in all enclosures (data not shown), indicating background levels less than 10^5^ viruses per ml as described previously in other Bergen based mesocosm experiments [12,13]. Although there was little variation in *E. huxleyi* genotypes throughout the experiment, the virus (EhV) that infects this alga did show considerable variation. DGGE analysis (Figure 4) indicated that the EhV population was more diverse and had a more variable genetic structure than the host. Whilst DGGE has its limitations due to co-migration events meaning that a single band can be comprised of >1 OTU, it is still accepted as a useful tool for looking at changes in microbial communities. In particular, DGGE has proven to be a robust and reliable technique for the study of EhVs. Limitations often described in the literature, centre on PCR-DGGE designed to target a large taxonomic group where the scale of diversity is massive, e.g., 16S rRNA. Focussing on a smaller taxonomic unit improves resolution [27] as does careful design and optimisation of primers. A two-stage PCR has been well optimised for EhV with the use of a GC-clamp to improve resolution. Although not all EhVs can be discriminated from one another, such as EhV-201 and EhV-205 that only differ by 1 bp in the target *mcp* region, virus isolates, including EhVs 203, 201, 202, 163, 84 and 86, can clearly be separated on a DGGE gel, with EhV-84 and EhV-86 differing from each other by only 3 bp [13]. Furthermore, DGGE gels for EhV have been found to be highly reproducible with the samples being run on >1 separate gels, generating the same migration profile. This is also corroborated by previous work of Sorensen et al. [14] and Martinez-Martinez et al. [12], where replicate gels routinely produce the same profile. 

**Figure 5:**
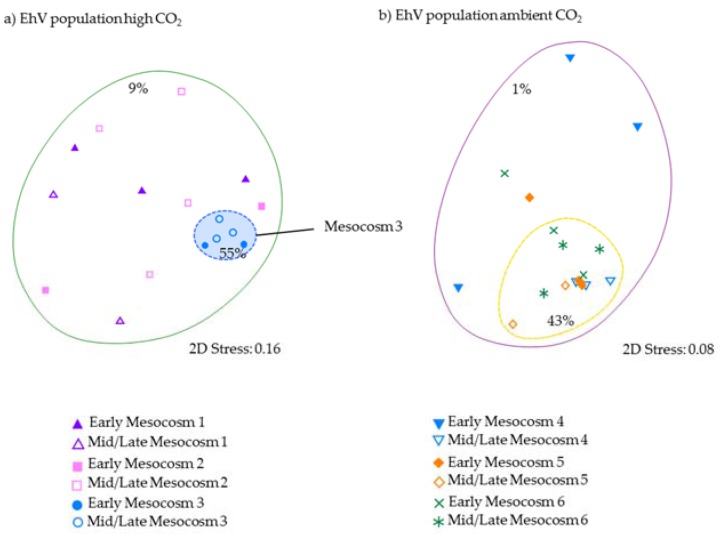
Bray–Curtis multidimensional plots based on the DGGE profiles (Figure 4) for EhV from (**a**) the high *p*CO_2_-treatment mesocosms 1, 2, and 3 and (**b**) ambient *p*CO_2_-treatment mesocosms 4, 5, and 6. “Early stage” corresponds to 7–9 May when coccolithophore numbers were <1000 cells mL^−1^ in ambient enclosures and “mid/late stage” corresponds to 12–14 May when coccolithophore numbers exceeded 1500 cells mL^−1^ in ambient enclosures. Contours indicate the percentage similarity, as indicated.

In the early stages of the mesocosm bloom development before 9 May, when coccolithophore numbers were <800 cells mL^−1^, there was high variability in the EhV population, both between replicates and on different days sampled, for example within mesocosm 4 on 7 May there were four bands, on 8 May seven bands and on 9 May eight bands, with four of these being unique; the percentage similarity was less than 40%. Whilst we cannot ascertain that each band represents a single out, we can still infer the changes observed and the overall temporal patterns of diversity. A Bray–Curtis similarity analysis (Figure 5) showed that, as coccolithophore numbers increased, exceeding 1.5 × 10^3^ cells mL^−1^ in ambient enclosures, there was less variability in the EhV population in the ambient *p*CO_2_-treatment mesocosms, which shared more similarity (>43%) between replicates. This was compared to as little as 1% in the early stages of the experiment when coccolithophore numbers were less than 1 × 10^3^ cells mL^−1^ (Figure 5).

Within the 2 high-*p*CO_2_ treatment enclosures that had the lowest coccolithophore cell number (M1 and M2), the DGGE profiles showed low similarity in the EhV population between dates and replicates (Figure 4). Stabilisation of the EhV population was not evident as the experiment proceeded in the high *p*CO_2_ samples, mesocosms 1 and 2, and similarity between samples remained low (9%). In contrast, in mesocosm M3, all samples shared at least 55% similarity, indicating a stable EhV population across the time series. One of the dominant bands on the DGGE profile in M3 (marked with a triangle in Figure 4a) was an EhV genotype that also dominated in the ambient-*p*CO_2_ treatments in enclosures M4, M5 and M6.

Samples from the 9 and 12 May from all six mesocosms were additionally run on the same gel. The shapes on the gels (Figure 4) indicate bands that migrated to the same position and hence can be inferred to be the same EhV sequence. 

## 4. Discussion

A number of mesocosm experiments have been performed to investigate the potential effects of increased *p*CO_2_ and reduced pH on complex pelagic assemblages—from bacteria to zooplankton. Mesocosm enclosures have the advantage of capturing more of the intrinsic complexity of a pelagic assemblage than is possible in a laboratory experiment because of the very large volumes of water (several thousand litres) that are involved. They also, by the nature of enclosure, eliminate problems of dispersal that make the study of variability in natural environments so complex. They are fundamentally attractive to experimentalists because they offer a means to manipulate large water volumes, with their associated planktonic assemblages, in order to test the effects of environmental problems such as eutrophication or ocean acidification.

In this study, we aimed to investigate how phytoplankton might respond in a future high-*p*CO_2_ ocean by comparing natural phytoplankton assemblage development in enclosures at ambient *p*CO_2_, (initial condition ca. 300 µatm.) and enriched *p*CO_2_ conditions (initially ca. 700 µatm.)—Figure 1a,b; see also Hopkins et al. [18]. During the course of the experiment, utilisation of CO_2_ by phytoplankton reduced *p*CO_2_ and the *p*CO_2_/pH values were continually changing. An obvious difference between the treatments was that less phytoplankton biomass, as indicted by chlorophyll *a* concentration, developed in the high- compared to the ambient-*p*CO_2_ conditions (Figure 1e). Not only did less biomass develop, but primary production (Figure 1f) was also lower under high-, rather than under ambient-*p*CO_2_, suggesting that increased *p*CO_2_ might have a deleterious effect on the total phytoplankton biomass. In contrast, other experiments have suggested that dissolved inorganic carbon uptake would be enhanced under elevated *p*CO_2_ conditions [28]. Different phytoplankton types are likely to respond differently to *p*CO_2_ and Riebesell et al. suggested that diatoms showed enhanced uptake, whereas coccolithophores did not [28]. There was no suggestion in that study, though, that coccolithophore growth might be reduced under elevated *p*CO_2_ conditions.

The dominant species of coccolithophore within the mesocosms was *E. huxleyi*, with coccolithophores being reported as contributing 6% and 12% to the total flagellate biomass for M1 and M6, respectively [18]. Manipulating the development of *E. huxleyi* blooms within mesocosm enclosures is well established at the mesocosm facility at Raunefjorden, and is well documented. Addition of nitrate and phosphate, with the omission of silicate, usually results in a bloom of *E. huxleyi* at this site, particularly during May/June [11,12,14] and an increase in *E. huxleyi* abundance happened in the present study. Previous mesocosm experiments of this nature have described a dominance of coccolithophores; however, differences in methodology most likely resulted in the dominance of large picoeukaryotes [18]. In the present study, a single nutrient enrichment was undertaken at the beginning of the study, whereas daily enrichments are often used e.g., Jacquet et al. [29]. Nevertheless, the maximum number of *E. huxleyi* cells that developed was significantly lower in the high- compared to ambient-*p*CO_2_ conditions (Figure 2), suggesting that *E. huxleyi* might be particularly susceptible to variations in *p*CO_2_. 

Replication between enclosures was rather variable. The peak chlorophyll concentrations were very similar in the three ambient *p*CO_2_ replicates (Figure 1e), but the bloom decayed more rapidly in M5 than in M4 and M6; there were similar differences in primary production (Figure 1f). However, the largest difference between replicates was in coccolithophore numbers (Figure 2), which showed significant differences between the three high-*p*CO_2_ treatment mesocosms with such differences between replicates in other key phytoplankton groups not reported by Hopkins et al. [18]. Enclosure M3 had coccolithophore cell numbers at the peak of the bloom that were very similar to the three ambient *p*CO_2_ mesocosms, unlike the lower numbers in M1 and M2. Although cell numbers were different in M3, no major differences in *E. huxleyi* genotype or phenotype were detected between treatments or over the course of the experiment, suggesting a stable community within all enclosures over the duration of the experiment. Stable *E. huxleyi* populations have been found in previous mesocosm experiments [12,14] and, indeed, in naturally occurring *E. huxleyi* blooms [9,17]. In this study, we have no evidence to support the hypothesis that higher *p*CO_2_ conditions might benefit certain *E. huxleyi* genotypes; we could detect no restructuring of the *E. huxleyi* population in the different *p*CO_2_ treatments.

Contradictory results are constant features of experiments to investigate the effect of *p*CO_2_/pH change on coccolithophores. In laboratory culture experiments, Riebesell et al. [30], Zondervan et al. [31] and Richier et al. [32] all reported increased production by *E. huxleyi* under elevated *p*CO_2_ conditions, but Sciandra et al. [33] and Langer et al. [4] found decreased production. In two different CO_2_-manipulated mesocosm experiments in the Raunefjorden, Engel et al. [6] and Paulino et al. [34] found little difference in *E. huxleyi* cell concentrations over the course of their experiments. However, Engel et al. [6] calculated that the net specific growth rate of *E. huxleyi* was reduced at 710 µatm compared with 410 μatm. In a long-term batch culture experiment conducted over one year, Lohbeck et al. [5] found that *E. huxleyi* cultures maintained at ambient *p*CO_2_ (400 μatm) went through 530 generations over the one year experimental period, but the same strain cultured at 1100 μatm achieved only 500 generations, and, at 2200 μatm, growth was even lower, with only 430 generations. *E. huxleyi* would appear to be more sensitive to *p*CO_2_ change than other phytoplankton. 

In the context of the present study, the reduced primary production in the high-*p*CO_2_ treatment enclosures is consistent with the finding of Lohbeck et al. [5] that higher *p*CO_2_/lower pH reduces primary production of an *E. huxleyi* dominated phytoplankton assemblage, although, in this study, other phytoplankton groups, e.g., picoeukaryotes, cryptophytes and cyanobacteria, will have also contributed to this. However, it is not consistent with the suggestion of Rivero-Calle et al. [8] that increasing *p*CO_2_ is one of the factors most responsible for the decadal increase in abundance of coccolithophores in the North Atlantic. The relationship between *p*CO_2_/pH change and success or, otherwise of cocccolithophores, remains confusing and requires clearer examination of mechanisms that might lead to phytoplankton changes in the future ocean.

Given that the coccolithophores numbers in enclosure M3 were very different from the other two high pCO_2_ treatments, and yet the *E. huxleyi* genetic diversity was very similar in all three enclosures, could viral infection by EhV be a contributing factor to explain the observed variations within and between treatments? In the three ambient-*p*CO_2_ enclosures, the EhV population followed a pattern that has been seen in other mesocosm experiments—high variability during the early stages of phytoplankton bloom development, with a smaller number of genotypes coming to dominate as the *E. huxleyi* numbers increase [12,14]. Although phytoplankton bloom development in the current study was short (<10 days), there was sufficient time for the virus populations to change because EhV populations are inherently dynamic [9] and known to change on very short time scales [14]. Daily changes in EhV composition can be expected since Sorensen et al. [14] showed that EhVs can appear/disappear from the water column in a matter of hours. 

In the high-*p*CO_2_ treatment enclosures M1 and M2, where *E. huxleyi* population densities did not exceed 1.1 × 10^3^ mL^−1^, the EhV population did not stabilise (Figure 4 and Figure 5) and EhV diversity was typical of early or non-bloom conditions [10]; that is, it was a highly dynamic and diverse EhV population. EhVs are known to have different host ranges [35] as well as different characteristics of infection, such as burst size and latent period. Therefore, any changes in environmental conditions that directly affect these traits could ultimately select for different genotypes, hence restructuring the EhV population. 

In contrast, the third high-*p*CO_2_ treatment enclosure M3, where coccolithophore cell densities reached similar values to ambient enclosures, and the EhV population structure was very different. Two EhVs dominated over the course of the experiment (Figure 4) and the population was much less changeable compared to the other high-*p*CO_2_ enclosures and, indeed, to the three ambient enclosures. The EhV assemblage in M3, right from the early stages of the experiment, reflected what would be expected in the later stages of a bloom. Even in the early stages of the bloom, the EhV assemblage was stable and clustered closely in the MDS plot (Figure 5) with samples taken later in the bloom.

Given that pH and *p*CO_2_ were so similar throughout the experiment in the three high-*p*CO_2_ enclosures, why did enclosure M3 have lower coccolithophore numbers and a lower and stable EhV diversity? Other studies have shown that, under non-bloom conditions, many different EhV genotypes are present and abundance fluctuates on short time scales [14]. In addition, during the initial phase of an *E. huxleyi* bloom, EhV populations remain diverse and are often highly dynamic. Sorensen et al. [14] showed that, as a bloom developed in a mesocosm experiment and viruses numbers proliferated, one viral genotype dominated, and suggested that this dominant virus caused the termination of the bloom. However, the dominant EhV is not always the same, even when host genotypes do not vary. Martínez-Martínez et al. [12] and Sorensen et al. [14] found that, although *E. huxleyi* populations were dominated by the same genotypes in different years at Raunefjorden (2000, 2003 and 2008), viruses changed and the dominant EhV in 2008 was different to the EhVs that dominated during the 2000 and 2003 mesocosm experiments. The reason is not known, but these authors suggest that a slight change in environmental conditions might have favoured dominance by a different virus genotype. Whilst the number of mesocosms sampled might be perceived as limited, the fact that Martinéz-Martinéz [35] described how the same DGGE profile was generated from four replicate mesocosms; in his studies, we can assume that the changes that we are observing are genuine.

Other mesocosm experiments have studied how altered *p*CO_2_ influences natural virus communities. Larsen et al. [36], using flow cytometry analysis and pulsed field gel electrophoresis (PFGE), found slightly more (but statistically insignificant) EhVs under present-day *p*CO_2_ mesocosms than in high-*p*CO_2_ treatments; this was not a consequence of lower *E. huxleyi* cell densities in the high-*p*CO_2_ treatments. The authors speculated that elevated-*p*CO_2_ may affect host–virus interactions or influence viral replication, since a 26 kb genome virus was only detected in ambient conditions, and was absent from high-*p*CO_2_ treatments, and a 105 kb genome virus was only detected in the highest *p*CO_2_ treatment of 1050 ppm. Unfortunately, the taxonomic affiliation of the viruses was not verified, which limits comparison with the present study.

Some laboratory experiments have investigated the effect of higher *p*CO_2_ on marine phytoplankton viruses. Carreira et al. [37] studied interaction between *E. huxleyi* and the virus EhV-99B1. *E. huxleyi* growth rate was not affected by the different *p*CO_2_ treatments, but the burst size of EhV-99B1 was lower in present-day, compared with higher and lower (pre-industrial) *p*CO_2_. In addition, release of EhVs was delayed in high-*p*CO_2_ treatments. Other virus groups that have also been tested for sensitivity to elevated *p*CO_2_. Chen et al. [38] found lower burst size of the *Phaeocystis globosa* virus (PgV) at high-*p*CO_2_, and Traving et al. [39] found that the cyanophage S-PM2, which infects *Synechococcus* sp, had reduced burst size at lower pH. However, the extracellular phase, quantified as infectivity loss rates/decay, did not change. These experiments illustrate that *p*CO_2_ can influence virus–host interactions, albeit to a relatively minor extent. However, extrapolation from these laboratory-scale experiments to natural virus communities would involve considerable uncertainty.

Another study considered a much longer time scale. Coolen [40] investigated *E. huxleyi* and EhV diversity in Black Sea sediments over a 7000 year period, showing that EhV diversity was highest during periods of change in hydrological and nutrient regimes. Shifts in EhV genotypic diversity typically coincided with Holocene environmental change with some viruses having limited persistence, yet others were found to persist for over a century. This study alluded to the impact that a change in CO_2_/pH could have on future EhV populations.

Might the different EhV populations that dominated in each enclosure be an explanation for the differences observed? It is generally accepted that virus infection is a major reason why *E. huxleyi* cells stop growing and blooms are terminated [14]. Certainly, nutrients were still available, albeit at low concentrations when biomass peaked in each enclosure (Figure 1c,d). If viral mortality was the major limit on bloom development, then enclosures M1 & M2 must have been infected with more aggressive EhVs than in the other treatments. The effect of *p*CO_2_ treatment, per se, on *E. huxleyi* cells cannot be responsible for the reduced growth in the two high-*p*CO_2_ treatments because an identical pH/pCO_2_ treatment in M3 did not reduce the size of the bloom. Therefore, *p*CO_2_ change must have resulted in different viral diversity, if infection is indeed the main cause of lower cell numbers and smaller *E. huxleyi* bloom.

How might reduced EhV diversity in M3 have resulted in higher coccolithophore numbers developing than in the two other high *p*CO_2_ treatments where growth was curtailed? One explanation would be that the EhVs that lead to rapid termination of *E. huxleyi* growth [14] were not present in sufficient numbers in M3 to suppress growth of the *E. huxleyi* population in this enclosure. Genetic diversity in natural populations of EhVs, especially in pre-bloom conditions [10], coupled with the rapid rate at which individual EhVs can come to dominate [9,14] means that the matrix of EhVs that could be selected for is large. It is not clear why only two EhVs were dominant in enclosure M3, but it is probably significant that viral infection in this enclosure did not suppress growth of *E. huxleyi* compared to M1 and M2.

In this study, it is difficult to distinguish whether *p*CO_2_ change is affecting the viruses specifically, or their hosts independently, and/or the interactions between them. Both the external virus population (the virus particles present in the water used to fill the mesocosms) and internal virus population (present in infected *E. huxleyi* cells) are important for the ultimate progression of the *E. huxleyi* bloom and EhV population. By comparing data from Schroeder et al. [13] and Martínez Martínez [12] it can be seen that the diversity of EhVs amplified from water samples within a mesocosm bloom can be very different from that amplified from *E. huxleyi* cells, particularly at the early stage of the bloom—that is, external and internal EhV assemblages can have very different composition. Thus, studies that aim to explain the effect of elevated *p*CO_2_ must incorporate into their experimental design ways to distinguish between a direct effect on external virus particles, or on *E. huxleyi* cells, or on EhVs that have already infected *E. huxleyi* cells. Alternatively, other unknown factors that are not related to *p*CO_2_ cannot be dismissed and might be the cause of the different coccolithophore response in enclosure M3.

Our study demonstrates the need for further investigations on the effects of elevated *p*CO_2_ on the *E. huxleyi* EhV system since there are ecological impacts of virus competition on biogeochemical cycles. Nissimov et al. [41] investigated competition between the two EhVs, finding that EhV-207 had a competitive advantage over EhV-86. It would thus be of value to determine how external factors, such as elevated CO_2_, would affect relative competitive ability of EhVs; would EhV-207 still outcompete EhV-86?

In this study, we have shown that elevated *p*CO_2_ can affect the structure of EhV assemblages. The data do not allow us to distinguish if this is a direct impact of *p*CO_2_ on the viruses themselves, but it is clear that caution is required in interpreting results from manipulation experiments and that deep analysis is required to truly understand how complex assemblages respond. For example, analysis of only the chlorophyll concentration or primary production data would not have revealed that there were differences in the triplicate high-*p*CO_2_ treatments: cell counts and microscopy would not have revealed the difference in *E. huxleyi* diversity in the replicate enclosures, and only analysis of virus genotypes could have revealed how different the viruses were in apparently identical replicate enclosures. We suggest that viruses cannot be ignored in any study of the potential effects of ocean acidification on phytoplankton productivity in the future high-CO_2_ ocean. 

## Supplementary Materials

The following are available online at www.mdpi.com/1999-4915/9/3/41/s1, Figure S1. DGGE image of *E. huxleyi* amplified *gpa*-PCR products.
