# Supplementary material for: Change in Emiliania huxleyi Virus Assemblage Diversity but Not in Host Genetic Composition during an Ocean Acidification Mesocosm Experiment
Source: Viruses. 2017 Mar 8;9(3):41. doi: 10.3390/v9030041 (PMC5371796; doi:10.3390/v9030041)
Supplement: Supplementary file 1 [file viruses-09-00041-s001.docx]

Supplementary Materials: Change in *Emiliania huxleyi* Virus Assemblage Diversity but Not in Its Host Genetic Composition during an Ocean Acidification Mesocosm Experiment

Andrea Highfield, Ian Joint, Jack A. Gilbert, Katharine J. Crawfurd and Declan C. Schroeder

1 2 3 4 5 6

1 2 3 4 5 6 1 2 3 4 5 6 6

High CO_2_

Ambient CO_2_ High CO_2_

Ambient CO_2_ High CO_2_

Ambient CO_2_

7 May

Treatment

Mesocosm

10 May


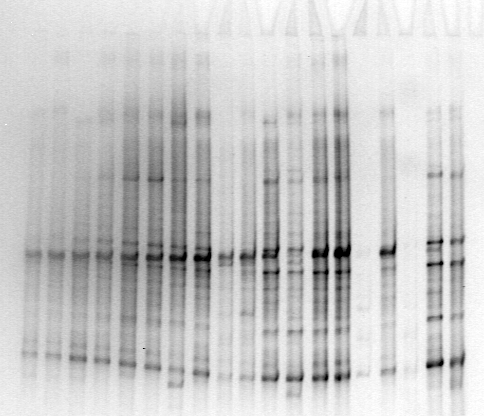


13 May

**Figure S1.** DGGE image of *E. huxleyi* amplified *gpa*-PCR products from (day 1) 9th, (day 4/5) 11th or 12th and (day 7/8) 13th or 14th May from mesocosms 1 to 6. Arrows indicate dominant bands detected in >70% of samples analysed.

© 2017 by the authors. Licensee MDPI, Basel, Switzerland. This article is an open access article distributed under the terms and conditions of the Creative Commons by Attribution (CC-BY) license (http://creativecommons.org/licenses/by/4.0/).
